# Formulated collagen gel accelerates healing rate immediately after application in patients with diabetic neuropathic foot ulcers

**DOI:** 10.1111/j.1524-475X.2011.00669.x

**Published:** 2011-05

**Authors:** Peter Blume, Vickie R Driver, Arthur J Tallis, Robert S Kirsner, Roy Kroeker, Wyatt G Payne, Soma Wali, William Marston, Cyaandi Dove, Robert L Engler, Lois A Chandler, Barbara K Sosnowski

**Affiliations:** 1 Affiliated Foot SurgeonsNew Haven, Connecticut; 2 Department of Surgery, Boston University Medical Center and School of MedicineBoston, Massachusetts; 3 Associated Foot and Ankle SpecialistsPhoenix, Arizona; 4 Department of Dermatology and Cutaneous Surgery, University of MiamiMiami, Florida; 5 Kroeker Podiatry ClinicFresno, California; 6 Institute of Tissue Repair, Regeneration and Rehabilitation, Bay Pines VA Health Care SystemBay Pines, Florida; 7 Department of Internal Medicine, Olive-View UCLA Medical CenterSylmar, California; 8 Department of Vascular Surgery, University of North CarolinaChapel Hill, North Carolina; 9 Advanced Foot and Ankle CenterLas Vegas, Nevada; 10 Department of Medicine, University of CaliforniaSan Diego, California; 11 Tissue Repair Company, Cardium TherapeuticsSan Diego, California

## Abstract

We assessed the safety and efficacy of Formulated Collagen Gel (FCG) alone and with Ad5PDGF-B (GAM501) compared with Standard of Care (SOC) in patients with 1.5–10.0 cm^2^ chronic diabetic neuropathic foot ulcers that healed <30% during Run-in. Wound size was assessed by planimetry of acetate tracings and photographs in 124 patients. Comparison of data sets revealed that acetate tracings frequently overestimated areas at some sites. For per-protocol analysis, 113 patients qualified using acetate tracings but only 82 qualified using photographs. Prior animal studies suggested that collagen alone would have little effect on healing and would serve as a negative control. Surprisingly trends for increased incidence of complete closure were observed for both GAM501 (41%) and FCG (45%) vs. Standard of Care (31%). By photographic data, Standard of Care had no significant effect on change in wound radius (mm/week) from during Run-in to Week 1 (−0.06±0.32 to 0.78±1.53, *p*=ns) but both FCG (−0.08±0.61 to 1.97±1.77, *p*<0.002) and GAM501 (−0.02±0.58 to 1.46±1.37, *p*<0.002) significantly increased healing rates that gradually declined over subsequent weeks. Both GAM501 and FCG appeared to be safe and well tolerated, and alternate dosing schedules hold promise to improve overall complete wound closure in adequately powered trials.

Approximately 24 million people in the US have diabetes and 800,000 new cases are identified each year.^1^ Many diabetic patients develop diabetic peripheral neuropathy. Among all diabetic patients 15% will eventually develop a Diabetic neuropathic Foot Ulcer (DFU), 25% of whom will have a foot amputation and subsequent 3-year survival rate of 50% despite currently available therapies.^2^,^3^ The current Standard of Care (SOC) for DFU includes surgical debridement, moist dressing changes, and off-loading.^4^ SOC treatment results in healing incidences of approximately 25% after 12 weeks and 30% after 20 weeks.^5^ In chronic DFU, the healing process is impaired in part due to deficiency of growth factors.^6^–^9^ Currently available secondary interventions include living skin equivalents (e.g., Apligraf, Organogenesis Inc., Canton, MA; Dermagraft, Advanced Biohealing Inc., Westport, CT), Becaplermin (Regranex, platelet-derived growth factor-B homodimer [PDGF-BB], Systagenix Wound Management, Gargrave, UK), hyperbaric oxygen, negative pressure devices, antibiotics for infection, and specialized dressings. These interventions provide moderate improvement over SOC, generally only 15–20%, and may be expensive and time consuming. For example Becaplermin is designed to be applied daily for up to 20 weeks due to short persistence of the growth factor protein in the wound.^6^ While exogenously applied growth factors can improve healing, a more convenient and less burdensome application method would be helpful, particularly since proper wound care and compliance are frequently problematic in diabetic patients. Delivery of a growth factor gene and transient protein expression at the site where it is needed could overcome the need for daily protein administration. In view of the continuing need for better therapeutics, particularly in the case of hard to treat wounds, Tissue Repair Company (a subsidiary of Cardium Therapeutics Inc., San Diego, CA) developed a Formulated Collagen Gel (FCG) and a replication-incompetent adenovirus serotype 5 (Ad5) vector. In preclinical wound healing models the PDGF-B gene inserted into the adenovector mixed with FCG was the most effective on indices of wound healing among a number of growth factor genes evaluated^10^,^11^ while FCG alone had little effect. FCG contains 2.6% bovine collagen in a specialized buffer providing protein stabilization and promotion of a balanced healing environment. FCG is believed to have several important attributes: it holds the Ad5 vector within the ulcer site by electrostatic attraction (positively charged collagen, negatively charged adenovector), it has binding sites for growth factors (i.e., PDGF-BB, 160 amino acid form), and it provides a scaffold for migration of cells responsible for generation of granulation tissue.^12^ In several preclinical models, application of FCG containing Ad5PDGF-B (GAM501) resulted in transfection of wound repair cells, provided sustained PDGF-BB production, and caused enhanced migration and proliferation of inflammatory cells, endothelial cells, fibroblasts, and other connective tissue cell types.^10^,^11^ This report describes the results of a Phase 2 blinded, controlled 12 week trial of three interventions (GAM501, FCG, and SOC) in diabetic patients with chronic, non-healing neuropathic ulcers.

## MATERIALS AND METHODS

The primary objective of the trial was to evaluate the safety and efficacy of GAM501 vs. FCG and SOC on the incidence of complete ulcer closure. The study was initially planned to enroll up to 210 patients and designed for a projected *alpha* of 0.05 and a *beta* (power) of 90% based on the phase 1/2 trial of GAM501 and historical SOC closure incidences of about 25–30%.^5^,^13^ Enrollment criteria included type 1 or 2 diabetic patients over age 18 with a Wagner Classification Grade 1 cutaneous lower extremity ulcer between 1.5 and 10.0 cm^2^ in area that had been present for at least 6 weeks. Patients had peripheral neuropathy (inability to perceive 10 g pressure using a Semmes-Weinstein 5.07 monofilament (North Coast Medical, Gilroy, CA) in the peri-ulcer area) and adequate blood flow (TcpO_2_ >40 mmHg or a toe pressure ≥40 mmHg). Exclusion criteria included HbA1c >12%, ulcers on the heel, cellulitis, biopsy positive for beta hemolytic streptococci or total bacterial load >1 × 10^6^ CFU/g tissue (treatment with local antibiotics once and re-biopsy was allowed), or a decrease in ulcer size of >30% from screening to Treatment Day 1 indicating an ulcer likely to heal with SOC.^5^ For complete criteria see http://clinicaltrials.gov/ct2/show/NCT00493051.

Twenty-two sites with institutional review board approval of the protocol, in compliance with the Declaration of Helsinki (as amended, Oct 2000), randomized patients. Following qualification and informed consent, patients underwent surgical debridement of the ulcer, biopsy for culture, clinical ulcer assessment, ulcer photograph, and ulcer size measurement (acetate tracing for planimetry) on Day −14 to start a screening 2-week Run-in period with SOC treatment. The primary data was the site-generated weekly acetate tracing of the wound edge faxed to a central laboratory for area measurement (Canfield, Fairfield, NJ); weekly ulcer photographs were archived at the same central laboratory as a back up if confirmation was needed. All patients wore a special off-loading orthopedic shoe (DH Walker; Ossur, Coconut Creek, FL) during the Run-in period and throughout the trial. On Day −3 repeat clinical ulcer assessment was performed and qualified patients were randomized into one of five treatment groups: (1) SOC, (2) FCG one application on Day 1, (3) FCG two applications on Days 1 and 29 (4 weeks), (4) GAM501 one application on Day 1, (5) GAM501 two applications on Days 1 and 29 (4 weeks). Randomization ratios were: 1 : 1 : 1 : 2 : 2.

The Day 1 visit consisted of surgical debridement of the ulcer if medically necessary, clinical assessment of the ulcer site, ulcer photograph, and ulcer size measurement by acetate tracing to confirm that the patient qualified. Study treatment was administered to the wound in the GAM501 and FCG groups by study personnel unblinded only to treatment vs. SOC (blinded to GAM501 vs. FCG), and the wound was covered and left undisturbed for one week. Principal Investigators remained completely blinded. Patients randomized to the SOC group continued with daily dressing changes. All patients were seen and assessed weekly until ulcer closure or week 12. Patients whose ulcer closed (complete epithelialization with no drainage) entered a 12 week follow-up phase to assess durability; non-healing patients exited the study at week 12. An independent Data and Safety Monitoring Board (DSMB) reviewed the data periodically.

When the trial had enrolled over half of the planned number of patients, the DSMB reported that there were no safety concerns but that enrollment was slower than projected. While completely blinded to treatment assignment, the company reviewed the slow patient recruitment rate, its long-term development plan, and blinded investigator reports of very rapid initial healing rates in some patients. Initial rapid healing rates in response to treatment with GAM501 had also been noted in the Phase 1/2 trial.^13^ Taking these factors into consideration, a decision was made to modify the trial design to examine initial healing rates as an additional primary end point, to combine the one- and two-dose treatment arms and reduce the combined study group sizes (and thus overall patient enrollment). Following this determination and before unblinding the trial, the Statistical Analysis Plan (SAP1) was written to combine the one- and two-dose treatment arms of both GAM501 and FCG, making the trial exploratory, since the initial healing rate would not be affected by the second treatment and the power to detect a treatment effect on initial healing rate would thus be increased. The SAP1 also specified that the rate of change of wound radius (wound-healing rate) over the first 4 weeks and the incidence of complete wound closure in the combined one and two dose groups were co-primary endpoints, and that the analysis would be per-protocol. The weekly healing rates were secondary endpoints. Early wound healing rates have been shown to predict eventual wound closure and are a variable that likely would be affected by an effective treatment compared with SOC.^14^ Note that the primary interest of the trial was to compare GAM501 to SOC and FCG as it had been assumed based on a number of prior animal studies that FCG alone would not have an effect and be a negative control.

As enrolled, 124 treated patients constituted the intention to treat (ITT) population included in the demographic and safety analyses. After database lock a blinded data review identified 11 patients with important protocol deviations who were not included in the Per-Protocol analysis (PP). Since the trial was exploratory the statistical analyses were perfomed PP on the resulting 113 patients.

During initial analyses, some major visual discrepancies in size and shape of the wound between acetate tracings and the corresponding photographs were noted. Details reported in the Results section justified an analysis using the photographs as the primary data source and a second exploratory Statistical Analysis Plan (SAP2) was finalized. The wound photographs had rulers included for calibration, and were taken from the same distance by means of a fixed focal length camera. Five blinded observers experienced or trained in wound evaluation independently traced all photographs from Day −14 through Week 8 using NIH Image J software. Using the coefficient of variation (SD/Mean) >0.1 as a guide, predefined rules for averaging the data from the five observers or repeating the measurements were included in SAP2. Areas for a few problematic photographs were determined by group consensus. Determination of complete wound closure by the sites was not changed.

### Data analysis

All data were entered into a web-based case report form. Wound radius used to track wound-healing rate was calculated as: *R*=SQRT(Area/π) and change in radius was expressed as mm/week. The total Run-in period varied widely so the healing rate during Run-in was calculated using the actual number of days/seven. Missing data were not imputed. Cumulative healing rates over multiple weeks (i.e. Day 1–Week 2, Day 1–Week 3, etc) were calculated from linear least squares fit for radius vs. time. Weekly healing rate was calculated as the difference in radius between sequential measurements. Change in radius over time more closely approximates tissue growth rates than area change.^15^

## RESULTS

A total of 129 patients were randomized; 124 patients qualified and were treated on Day 1 and constitute the ITT population. Demographic data, ulcer size and duration for the ITT population were similar between groups (Table 1). The predominant ulcer location was plantar in 89% of SOC, 88% of FCG, and 78% of GAM501 patients.

**Table 1:** Demographic characteristics and patient disposition

Variable	GAM501 Combined (*N*=72)	FCG Combined (*N*=33)	Standard of Care (*N*=19)
Age (years) Mean (SD)	57.9 (10.9)	56.2 (12.0)	54.8 (12.3)
Median (range)	60 (31–77)	59 (30–83)	51 (38–86)
Gender [*n* (%)]			
Male	50 (69%)	25 (76%)	15 (79%)
Female	22 (31%)	8 (24%)	4 (21%)
Caucasian	46 (64%)	21 (64%)	12 (63%)
Black or African American	10 (14%)	4 (12%)	2 (11%)
Hispanic	16 (22%)	8 (24%)	4 (21%)
American Indian or Alaskan Native	0 (0%)	0 (0%)	1 (5%)
Baseline ulcer size mean ± SD,cm^2^	3.1 ± 1.7	2.9 ± 1.1	2.8 ± 1.3
Ulcer duration mean ± SD, Months	18.4 ± 28.6	17.1 ± 26.8	11.6 ± 12.0
Type 1 diabetes (% in group)	6 (8%)	2 (6%)	2 (11%)
Type 2 diabetes	63 (88%)	29 (88%)	16 (84%)
Unspecified type of diabetes	3 (4%)	2 (6%)	1 (5%)
Diabetes duration (mean, years)	15	14	13
Hb A1c % (SD)	8.06 (1.82)	8.07 (1.45)	7.85 (1.34)
BMI, mean (SD)	33.70 (7.54)	33.08 (7.13)	34.15 (7.18)
BMI, median (range)	32.2 (22.2–66.6)	32.3 (20.7–50.2)	34.2 (23.7–49.4)
Completed the study	67 (92%)	31 (84%)	18 (95%)
Withdrew from the study	5 (7%)	2 (5%)	1 (5)
Adverse event	0 (0%)	2 (5%)	0 (0%)
Patient noncompliance	0 (0%)	0 (0%)	1 (5%)
Lost to follow-up	5 (7%)	0 (0%)	0 (0%)

No significant differences between groups for any parameter.

Review of the data by the DSMB found GAM501 and FCG to be safe in the ITT population; no adverse events were classified by the Investigators as likely or definitely related to treatment.

Of the 124 patients treated, 116 completed the study and eight were withdrawn (Table 1). For SAP1 the blinded data review identified 11 patients with protocol deviations for inclusion/exclusion criteria not met and treatment or visit non-compliance leaving 113 patients in the PP population. A 10% variation was allowed so that minimum wound size for inclusion was 1.35 cm^2^ and maximum allowable decrease from Day −14 to Day 1 was 33%.

Ulcer closure incidences were 5/16 (31%) in SOC, 14/31 (45%) in FCG, and 27/66 (41%) in GAM501, a non-significant trend. Using acetate data, there were no significant differences in wound radius healing rates from Day 1 to Week 4 between groups (data not shown). The finding that GAM501 and FCG had nearly identical effects was surprising as several non-diabetic and diabetic preclinical models had found GAM501 to be superior.^10^,^11^,^16^ To investigate this observation the wound photographs and acetate tracings were printed for visual comparison; the striking differences noted between the acetate tracing and corresponding photograph on Day 1 from some sites led to blinded wound photograph analysis as primary data using SAP2.

The results from the blinded photographic data review were unexpected. Wound area by photograph on Day 1 was less than 1.35 cm^2^ in 33 out of 133 patients (29%) and 10 patients had wound size decreases of greater than 33% during Run-in; eight patients met both exclusion criteria, making a total of 35 patients (31%) that likely should have been excluded from enrollment on Day 1. An additional patient was excluded because the wound covered a curved surface and area by photograph could not be accurately determined. Five patients excluded from SAP1 were found to qualify for SAP2, leaving a total of 82 patients for analysis in SAP2.

The mean wound size determined by acetate and photograph for the SAP2-excluded and—included patients through Week 2 is shown in Table 2. On Day 1 the difference between acetate and photographic areas was 163% for excluded patients and 20% for included patients (*p*<0.001). The data revealed systematically greater area measurements with acetate tracings compared with photographs with differences that were very large on Day 1 and much less thereafter. Exclusions were clustered among a few sites: six sites had 26 of 43 (60%) patients excluded in SAP2; the remaining 14 sites had 8 of 74 patients (11%) excluded in SAP2.

**Table 2:** Photographic and acetate wound size data

Time	Excluded Acetate	Excluded Photograph	Included Acetate	Included Photograph
Day 1	2.31 ± 0.78	0.90 ± 0.39*	3.38 ± 1.82	2.85 ± 1.57†
Week 1	0.91 ± 0.74	0.65 ± 0.54†	2.28 ± 1.78	2.17 ± 1.65
Week 2	0.78 ± 1.03	0.62 ± 0.55	1.96 ± 1.70	1.96 ± 1.64

All data cm^2^.

* *p*<0.001 vs. acetate,

† *p*<0.05 vs. acetate, paired *t* test. Mean ± S.D.

The 12-week complete closure incidences in SAP2 were: SOC: 4/13 (31%), FCG: 6/17 (35%), and GAM501: 21/51 (41%), *p*=ns. Note that the complete closure incidence for patients excluded using photographs as primary data in SAP2 was 17 of 35 (49%) suggesting that these small and/or rapidly closing wounds were likely to close without intervention as reported in published literature.^5^

Table 3 (SAP2) shows the cumulative wound healing rates for Run-in and for Day 1 through Week 4. Pair-wise comparisons between the three groups by ANOVA found that the only significant difference was between FCG and SOC for Day1–Week1 and Day1–Week2. Within each group all subsequent healing rates were compared with Run-in by paired *t*-tests. In the SOC group there was no significant change in healing rate from Run-in until Day1–Week2. In the FCG and GAM501 groups, all cumulative healing rates were significantly different than Run-in.

**Table 3:** Cumulative wound healing rates, decrease in radius in mm/week

Time period	SOC	FCG	GAM501
Run-in (Day14–Day 1)	−0.06 ± 0.32	−0.08 ± 0.61	−0.02 ± 0.58
Day 1–Week 1	0.78 ± 1.53	1.97 ± 1.77*,‡	1.46 ± 1.37‡
Day 1–Week 2	0.63 ± 0.71†	1.37 ± 0.93*,‡	1.06 ± 0.92‡
Day 1–Week 3	0.63 ± 0.57†	1.00 ± 0.66‡	0.83 ± 0.73‡
Day 1–Week 4	0.52 ± 0.58†	0.80 ± 0.52‡	0.73 ± 0.58‡

Positive number indicates radius decrease. Between Groups Test:

* *p*<0.05 compared with SOC by one way ANOVA; Within Group Tests: compared with Run-in:

† *p*<0.05.

‡ *p*<0.002. Mean ± SD.

We reasoned that the application of a wound healing adjuvant should increase the healing rate promptly after application and have a time-limited effect. Therefore, we tested whether the weekly healing rate was different for the 4 weeks after treatment than during Run-in for each group. Results in Table 4 from SAP2 show that Run-in healing rates for all groups were essentially zero. The weekly healing rates for SOC were never statistically different than zero. The GAM501 weekly rate was significantly non-zero through Week 4 and the FCG rate was significantly non-zero through Week 2. Table 4 also shows the statistical results for change in healing rate from Run-in. In the SOC group only the Week 2–Week 3 rate was increased over baseline. In the FCG group, the first 2 weeks were significantly increased from baseline. In the GAM501 groups, all subsequent healing rates were significantly increased over Run-in.

**Table 4:** Weekly wound healing rates; decrease in radius in mm/week from run-in through week 4, SAP2

Time period	SOC	FCG	GAM501
Run in	−0.06 ± 0.32	−0.08 ± 0.61	−0.02 ± 0.58
Day1–Week1	0.78 ± 1.53	1.97 ± 1.77*,†	1.46 ± 1.37*,†
Week1–Week2	0.48 ± 0.90	0.81 ± 0.85*,†	0.63 ± 1.05*,†
Week2–Week3	0.68 ± 1.16‡	0.29 ± 0.83	0.44 ± 0.89*,†
Week3–Week4	0.01 ± 0.94	0.34 ± 1.14	0.72 ± 1.29*,†

* *p*<0.001 from 0.0 rate of change, *t* test.

† *p*<0.01 from Run-in.

‡ *p*<0.05 from Run-in, *t*-test. Mean ± SD.

## DISCUSSION

The demographics of our patient population are similar to what has been reported in previous studies of neuropathic Wagner Class 1 DFUs, and there were no differences between groups. Entry criteria excluded patients with small wounds or with wounds decreasing in area more than 30% in a 2 week screening period; such wounds are unreliable for testing interventions as they tend to heal with SOC.^5^ Indeed our data showed a 49% complete closure incidence in the patients who had wound sizes <1.35 cm^2^ or closure rates >33% during Run-in by photographic analysis. Since the entry criteria included a requirement for chronic non-closure it may seem surprising that some patients were excluded during Run-in (five by acetate and 35 by photographic data). This may have been due to better SOC treatment and use of an off-loading boot during the Run-in period.

In the primary SAP1 analysis the wound closure incidence in SOC of 31% is slightly greater than the published 12 week incidence of ∼25%.^5^ The supportive care of weekly visits and encouraged compliance to off-loading may have had a positive effect. The numerically greater complete closure incidence over SOC observed in the GAM501 and FCG groups was not statistically significant; whether the lack of significance was due to small sample size could be determined by future studies. Despite the small group sizes, the fact that both GAM501 and FCG generated similar improvements in wound healing rates and complete closure incidence was surprising based on preclinical studies which suggested that FCG alone would not substantially increase the incidence of healing or complete wound closure, and thus serve as an additional control group. One possible explanation is that the patients had more bleeding at the time of treatment due to debridement than in the preclinical models, where repeat debridement was not performed before treatment. One limitation of our study is that we did not measure PDGF expression or duration in the patient's wounds. However, in preclinical studies PDGF expression in response to GAM501 persisted for about 1 week.^10^,^11^ Collagen (as used in both GAM501 and FCG) is known to activate platelets and release PDGF.^17^ We confirmed in vitro that exposure of human platelets to FCG triggers PDGF release. Thus the FCG group might also have augmented PDGF at the wound site to promote a wound healing response; defining the mechanisms of action of FCG is worthy of further study.

The results obtained from analysis of photographs during Run-in were quite disparate from the acetate tracings. The finding that 35 of 113 (31%) patients were excluded when the primary data was photographs is noteworthy. Acetate tracings have traditionally been used for wound measurements, as they are easy to perform and can conform to curved foot surfaces. Their disadvantages include blood in and around the wound or spreading on the acetate obscuring the wound edge, slippage of the acetate during tracing, and differing tracing techniques. In the current study, investigators and study coordinators were trained extensively and very detailed procedures were to be followed. Despite this effort, findings from the post hoc comparison of acetate- and photograph-based measurements revealed significant discrepancies and provide important lessons for future trials. Planimetry of acetate tracings gave systematically larger areas than planimetry of photographs (Table 2). The comparison also suggests a systematic overestimation on Day 1 which seemed to be clustered at a few sites. There are several possible explanations. The investigators at these sites may have been more aggressive in wound debridement causing more wound bleeding to spread on the acetate and thereby making the wound appear larger. It is also possible that enthusiasm for enrolling patients for a possibly beneficial treatment led to unconscious bias at the critical entry determination. One of the important lessons from our trial is that a more highly standardized and accurate method of sizing wounds is needed. While photographs lack the ability to eliminate curvature as a variable, they do lead to more objective and verifiable data. If acetate tracings are to be used as primary data, a quality control mechanism, likely using photographs, should be employed during trial execution. Recently available photographic equipment with laser scanning to correct for wound surface curvature may provide more accurate and reproducible data.

When we analyzed the patient data in SAP2, we again found a trend for improved complete closure of wounds for GAM501 and FCG compared with SOC, however, with only 82 patients the statistical power may have been insufficient. In contrast to the insignificant rates of change of wound radius using acetate data, which was likely biased by the apparent large errors over-estimating wound size measurements on Day 1, with photographic data there were significant differences. Pair-wise comparison between groups showed that FCG was significantly better than SOC for the time periods of Day1–Week1 and Day1–Week2 (Table 3). Within each group there was an immediate large and highly significant increase in cumulative wound-healing rate from Run-in to Week-1 that remained significant but decreased numerically at all subsequent time periods for both GAM501 and FCG. In contrast, the SOC group did not show a statistical increase in cumulative healing rate until Week 2, and it remained fairly constant. This comparison suggests a time-dependent effect of both GAM501 and FCG on wound-healing rate that appears to be different than SOC. Further insight is gained from the individual weekly wound healing rates. Compared with Run-in, the weekly healing rate only changed significantly in the SOC group at Week 2–3; the only interventions being weekly visits and encouraging the use of off-loading. In contrast, for both the GAM501 and FCG groups the healing rate increased markedly during Week 1 and then declined slowly over the next 3 weeks (Table 4). Thus, both GAM501 and FCG appeared to cause a large and rapid time-dependent effect on tissue growth rates. The study as completed was underpowered to detect any difference between the two interventions, which will be an important question for future trials. The study protocol limited treatment with GAM501 and FCG to one or two treatments. Based on the safety data and the timing of significant improvement in healing rates observed in this study, multiple treatments hold promise to further increase overall complete wound closure in future adequately powered trials.

We conclude from this exploratory trial that a single application of GAM501 or FCG increases the healing rate of neuropathic DFUs for the first two weeks after treatment; whereas SOC with weekly visits seems to have a much smaller and delayed effect on wound healing rate. This suggests that more frequent applications of GAM501 or FCG hold promise to significantly improve overall incidence of complete wound closure. Whether one or more administrations of GAM501 has advantages over FCG alone in certain circumstances, such as larger or more difficult to treat wounds, will require testing in future trials.

## Abbreviations

Ad5: Adenovirus serotype 5 vector
Ad5PDGF-B: E1-deleted adenovirus serotype 5 encoding human platelet-derived growth factor-B
CFU: Colony forming unit
DFU: Diabetic foot ulcer
DSMB: Data and safety monitoring board
FCG: Formulated Collagen Gel
GAM501: Gene Activated Matrix 501, a proprietary product
ITT: Intention to treat
PDGF-B: Platelet derived growth factor B gene
PDGF-BB: Platelet derived growth factor BB
PP: Per-protocol
SAP: Statistical analysis plan
SOC: Standard of Care

## Supporting Information

**Appendix S1.** The following principal investigators and institutions enrolled patients.
