# Supplementary material for: Formulated collagen gel accelerates healing rate immediately after application in patients with diabetic neuropathic foot ulcers
Source: Wound Repair Regen. 2011 May;19(3):302–8. doi: 10.1111/j.1524-475X.2011.00669.x (PMC3443373; doi:10.1111/j.1524-475X.2011.00669.x)
Supplement: Supplementary file 1 [file wrr0019-0302-SD1.doc]

**Appendix:** The following principle investigators and institutions enrolled patients:

Peter Bloom, DPM, Affiliated Foot Surgeons, New Haven, CT; Vickie R. Driver, DPM, Boston University Medical Center and School of Medicine, Boston, MA; Arthur J. Tallis, DPM, Associated Foot and Ankle Specialists, Phoenix, AZ; Robert S. Kirsner, MD, PhD, University of Miami, Miami, FL; Roy Kroeker, DPM, Fresno, CA; Wyatt Payne, MD, Bay Pines VAHCS, Bay Pines, FL; Soma Wali, MD, Olive View-UCLA Medical Center, Sylmar, CA; William Marston, MD, U. of North Carolina, Chapel Hill, NC; Cyaandi Dove, DPM, Advanced Foot and Ankle Center, Las Vagas, NV; Barbara Aung, DPM, Aung Foothealth Clinics, Tucson, AZ; David Abdoo, DPM, Foot Doctors of Watsonville, Watsonville, CA; Warren Garner, MD, LAC & USC Medical Center, Los Angeles, CA; Ian Gordon, MD, Long Beach VA Heath Care System, Long Beach, CA; Maria Guidry, MD, Univ. Texas Medical Branch, Galveston, TX; Gabriel Halperin, DPM, Innovative Medical Technologies, Los Angeles, CA; Scott Lipkin, DPM, Lehigh Valley Hospital, Allentown, PA; James Longobardi, DPM, Absolute Foot Care, Chula Vista, CA; Thomas Serena, MD, Penn North Centers for Advance Wound Care, Warren, PA; Bhavesh Shah, DPM, South Texas Foot Clinic, San Antonio, TX; Mark Starling, MD, Banner Baywood Medical Center, Mesa, AZ; Hans Moosa, MD, Vascular and Hand Surgery, Belleville, IL; Terry Treadwell, MD, Institute for Advanced Wound Care, Montgomery, AL
